# Use of pumice stone and silica fume as precursor material for the design of a geopolymer

**DOI:** 10.12688/f1000research.147701.2

**Published:** 2024-08-12

**Authors:** Alexis Iván Andrade Valle, Tito Oswaldo Castillo Campoverde, Cristian Andrés Marcillo Zapata, María Gabriela Zúñiga Rodríguez, Andrea Natalí Zárate Villacrés, Marcelo David Guerra Valladares, Mayte Lisbeth Mieles Mariño, Jefferson Javier Castillo Cevallos

**Affiliations:** 1 Engineering, Universitat Politecnica de Valencia, Valencia, Valencian Community, 46022, Spain; 2 Engineering, Universidad Nacional de Chimborazo, Riobamba, Chimborazo Province, 060150, Ecuador

**Keywords:** Alkali activation, molar concentration, geopolymer, silica fume, pumice powder

## Abstract

**Background:**

Geopolymers are alternative materials to cement because they require less energy in their production process; hence, they contribute to the reduction in CO _2_ emissions. This study aims to evaluate the possibility of using industrial residues such as silica fume (SF) to improve the physical and mechanical properties of a pumice stone (PS)-based geopolymer.

**Methods:**

Through an experimental methodology, the process starts with the extraction, grinding, and sieving of the raw material to carry out the physical and chemical characterization of the resulting material, followed by the dosage of the geopolymer mixture considering the factors that influence the resistance mechanical strength. Finally, the physical and mechanical properties of the geopolymer were characterized. This research was carried out in four stages: characterization of the pumice stone, design of the geopolymer through laboratory tests, application according to the dosage of the concrete, and analysis of the data through a multi-criteria analysis.

**Results:**

It was determined that the optimal percentage of SF replacement is 10%, which to improves the properties of the geopolymer allowing to reach a maximum resistance to compression and flexion of 14.10 MPa and 4.78 MPa respectively, showing that there is a direct relationship between the percentage of SF and the resistance.

**Conclusions:**

Geopolymer preparation involves the use of PS powder with a composition rich in silicon and aluminum. The factors influencing strength include the ratio of sodium silicate to sodium hydroxide, water content, temperature, curing time, molarity of sodium hydroxide, and binder ratio. The results showed an increase in the compression and flexural strength with 10% SF replacement. The geopolymer’s maximum compressive strength indicates its non-structural use, but it can be improved by reducing the PS powder size.

## Introduction

Concrete is the most commonly used construction material in civil engineering applications owing to its physical-mechanical properties, durability, and cost. These advantages compared to other materials imply an increase in the consumption of cement, whose industry is responsible for 8% of CO _2_ emissions worldwide according to Ref. 1. This is due to the significant amount of energy required to produce clinker, which is its main component. The Environmental Investigation Agency (EIA) ^2^ states that strategies must be adopted to optimize the use of cement, such as energy efficiency techniques, changing fuels to those with less carbon, promoting technological innovations, and promoting the efficiency of materials, the latter to reduce the clinker-cement production ratio and its total demand. The EIA also emphasizes that the incorporation of alternative binder materials could be key to reducing emissions from cement production.

Although alternatives for reducing CO _2_ emissions in cement production are growing, their large-scale implementation faces many challenges. These include research costs, development and adaptation of new technologies, and the need to modify existing regulations and standards. In addition, it is essential to involve all actors in the value chain, from cement producers to builders and end users, to ensure the transition to more sustainable construction. In this sense, education and awareness are key elements to promote adopting more sustainable practices in the construction sector. ^3^

Therefore, a new alternative that reduces the use of cement is geopolymers, which are obtained by alkaline activation of materials rich in alumina and silica, such as natural pozzolans, fly ash, and other industrial wastes. ^4^

References 5, 6, and 7 established that materials of volcanic origin favor alkaline activation due to the presence of aluminum silicates. The study seeks to establish whether the material of volcanic origin presents the necessary conditions to act as a precursor in the design of geopolymers.

Regarding the use of natural pozzolans such as pumice stone (PS) in the production of geopolymers, ^8^^,^^9^ it can be concluded that PS in alkaline-activated geopolymers provides satisfactory compressive strength at 28 days of 12 MPa. Furthermore, Ref. 8 determined that it is possible to use geopolymers with structural and non-structural applications that can reach a compressive strength of up to 24 MPa at 28 days.

Reference 10 explored the deposits throughout Ecuador, the largest found in the provinces of Tungurahua, Cotopaxi, and Ibarra. A quantitative and qualitative microscopic analysis determined that the material presents amorphous in its constitution, with a high presence of aluminum silicates, which is the base requirement of the research.

Regarding the use of industrial waste, research has focused on the use of fly ash; however, other industrial wastes, such as silica fume (SF), have great potential as precursor materials. From SF it has been shown that its incorporation into geopolymers at 2% by weight means an increase in compressive strength, while at 4% it would decrease it according to Refs. 11, 12, however, Ref. 13 refers to SF incorporations of up to 5% to obtain satisfactory resistance results. On the other hand, ^14^ concluded that up to 7% SF addition by weight increased the mechanical properties of the geopolymer, although an increase in the % reduced the resistance.

Therefore, the aim of this study was to characterize the physical and chemical properties of PS to produce a geopolymer with SF alkaline-activated with a solution of sodium silicate and sodium hydroxide at different concentrations.

## Methods

In the present investigation a combination of Pumice Stone (obtained from the Cotopaxi deposit, specifically from the “PROFUTURO” quarry), and Silica Fume (obtained from the company “Ferrekret” in Guayaquil) was used as precursor material in replacement percentages of 2.5%, 5% and 7.5%. Moreover, the precursor material used contained approximately 86% of particles of a size smaller than 0.3 mm and 25% smaller than 0.075 mm.

A combination of two alkaline solutions was used as activators: sodium silicate ( ${\mathrm{Na}}_{2}\mathrm{Si}O_{3}$ ) and sodium hydroxide ( NaOH ) in a ratio of 2.5. The concentration of these compounds in the aqueous solution was 8 Mol/l and 12 Mol/l. The reactive used to prepare the activating solutions were ${\mathrm{Na}}_{2}\mathrm{Si}O_{3}$ solution of 97% purity ( $11\%Na_{2}O,29.5\%\mathrm{Si}O_{2}$ ); NaOH in the form of flakes of 97% purity ( 97%NaOH,0.005%Ca,0.001%Fe ). These were supplied by the RELUBQUIM company.

The mixtures were prepared by mixing the precursor material (combination of Pumice Stone with Silica Fume) and the different activating solutions (varying their molar concentration). The alkaline binder solution ratio was maintained constant and equal to 0.75 (where the alkaline solution includes distilled water and the chemical compounds: Sodium hydroxide NaOH and Sodium silicate Na _2_SiO _3_). The specimens obtained were transported to an oven at 60°C to begin to cure, here they were kept for 120 hours. To proceed to a second curing stage, the specimens were covered with a plastic bag and kept until the testing day at room temperature and humidity (approximately 18°C and relative humidity of 50%).

**Figure 1.**
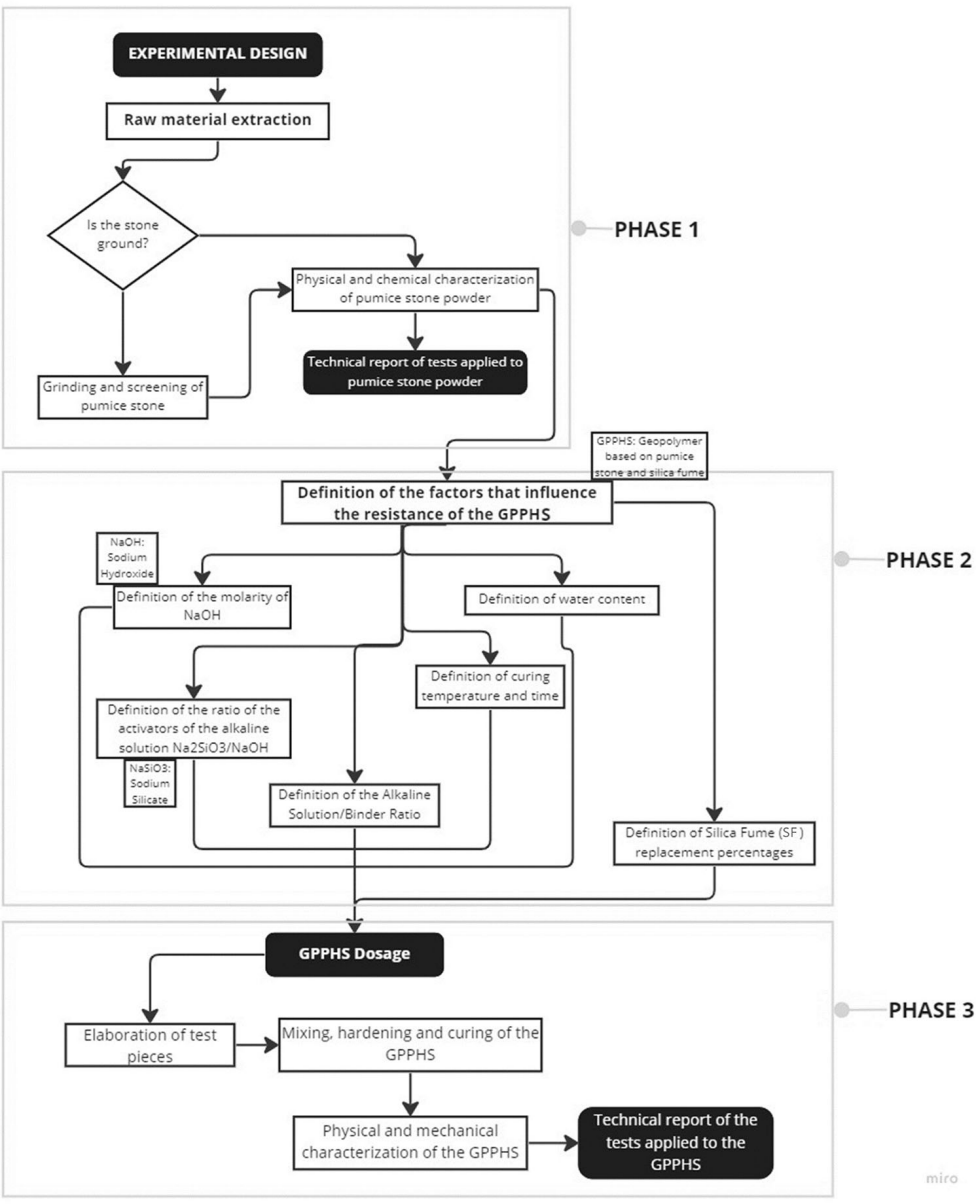
Methodology diagram materials.

Once the curing time had elapsed, the 40×40×160 mm prismatic specimens and 50×50×50 mm cubic specimens were tested to determine their flexural and compressive strength, respectively, for which the mortar standards ^15^^,^^16^ were used, in addition, for the physical characterization of the mixtures (density and absorption), the Ref. 17 standard was used. The specimens after 7 days of curing were subjected to mechanical tests, while after 28 days of curing, the samples were subjected to both physical and mechanical tests ( Figure 1).

### Pumice Stone (PS)

**Figure 2.**
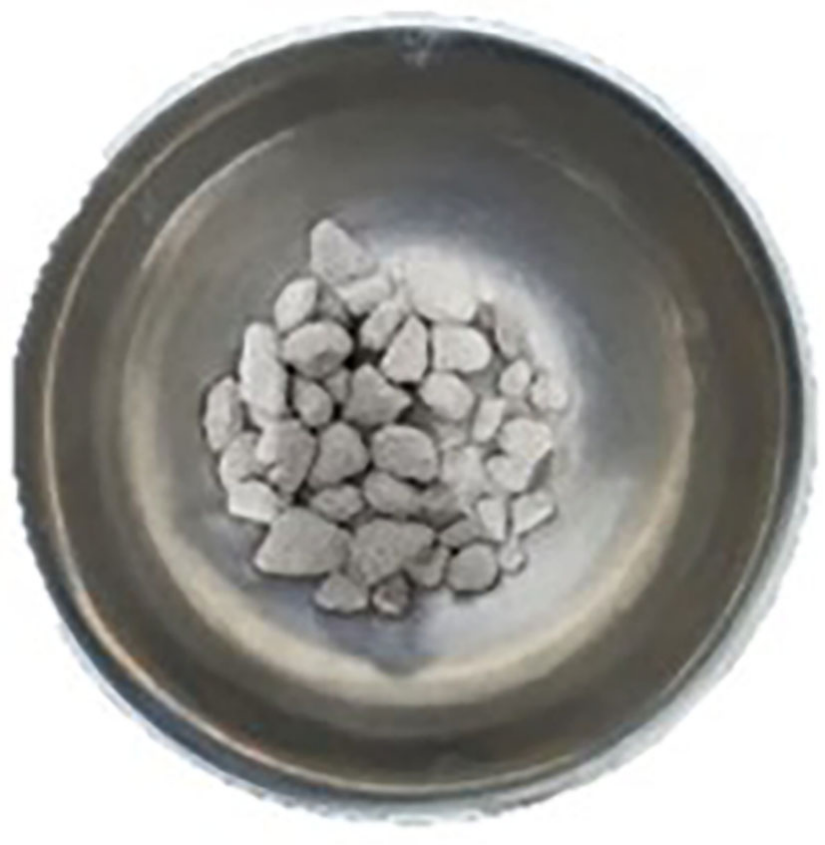
Extracted pumice stone.

The PS was obtained from the “Profuturo” mining area with code “201004,” located in the city of Latacunga, Cotopaxi province, with UTM coordinates 764747.34 m E, 9893327. 94 m S, and 2760 m elevation. See Figure 2.

### Silica fume

**Figure 3.**
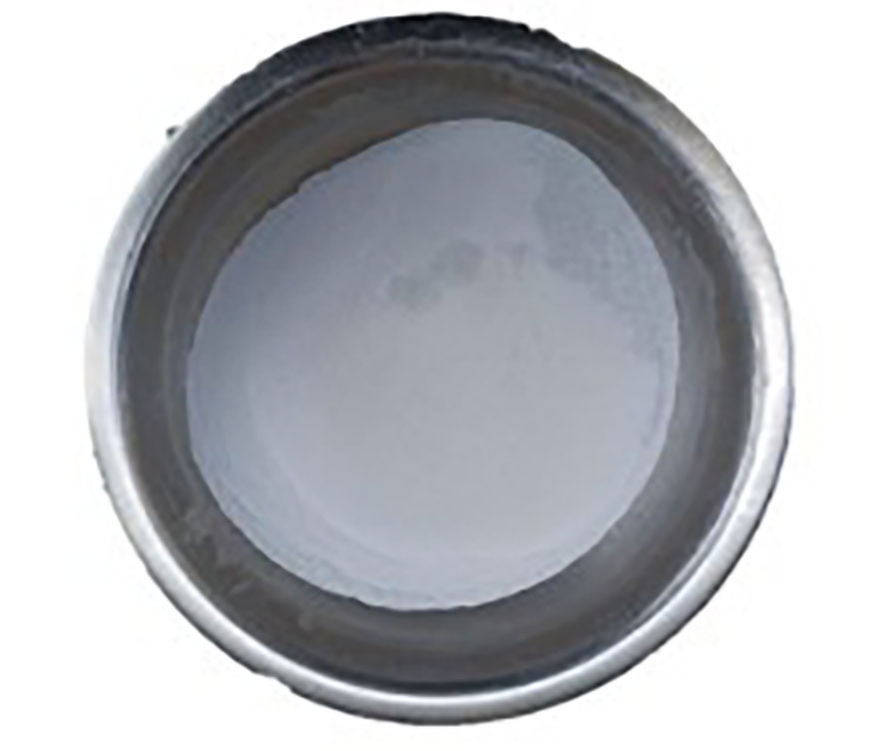
Silica fume (SF).

This industrial waste ( Figure 3) was purchased from the company “Ferrekret” located in the city of Guayaquil, in a presentation of 5 kg, and was stored in the warehouses of the UNACH Civil Engineering Laboratories in a cool and ventilated environment. Table 1 shows the chemical composition of silica fumes.

**Table 1.** SF chemical composition (Chemical composition of “Ferrekret”).

Component	Unit	Composition
SiO _2_ _	%w/w	91.10
C free	%w/w	-
Sic	%w/w	-
MgO	%w/w	0.27
Fe _2_O _3_ _	%w/w	0.13
Al _2_O _3_ _	%w/w	6.82
CaO	%w/w	0.47
Na _2_O _ _	%w/w	0.96
K _2_O _ _	%w/w	0.34

### Sodium silicate

This chemical substance was purchased from the company “Relubquim,” located in the city of Quito. It was stored in the warehouses of UNACH Civil Engineering Laboratories, always guaranteeing a cool, ventilated, and dark environment to avoid evaporation. The technical specifications of Sodium Silicate are shown in Table 2.

**Table 2.** Technical specifications of Na _2_ SiO _3_.

Parameter	Units	Specifications
Alkalinity	%Na _2_O	9.2% - 11%
Silicon dioxide	%SiO _2_	27.5% - 29.5%
Relationship	SiO _2_/Na _2_O	2.9 – 3.2
Concentration	°Be	41-43
Total solids	%m/m	-

### Sodium hydroxide

**Figure 4.**
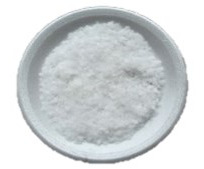
Sodium hydroxide.

This chemical substance was purchased from the company “Largo Rivera Herwin Roger-Novachem del Ecuador” in Quito. The sodium hydroxide used was solid in the form of flakes/lentils and was stored in the warehouse of the UNACH Environmental Engineering Laboratories, always guaranteeing a cool, ventilated, and dark environment to avoid reaction. See Figure 4. The technical specifications of Sodium Silicate are shown in Table 3.

**Table 3.** Technical specifications of NaOH (Certificate of analysis of “Largo Rivera Herwin Roger – No vachem del Ecuador”).

Result name	Unit	Specifications
Appearance		Report
NaOH	%	≥97.0
Calcium	%	≤0.005
Chloride	%	≤0.005
Heavy metals (as Ag)	%	≤0.002
Iron (Fe)	%	≤0.001
Magnesium	%	≤0.002
Mercury (Hg)	ppm	≤0.1
Nickel (Ni)	%	≤0.001
Compound nitrogen	%	≤0.001
Phosphate (PO4)	%	≤0.001
Potassium (K)	%	≤0.02
Sodium carbonate	%	≤1

### Physical and chemical characterization of pumice stone powder

To physically and chemically characterize the pumice powder, the raw material was extracted with the help of a hammer tree mill, rotary drum, and a 0.6 mm diameter sieve for its mesh.

**Physical characterization of pumice powder**

One of the determined physical properties of the PS powder is the oven-dry density and absorption under ^18^ procedures. This test began with the sample drying at a temperature of 110°C ± 5°C until obtaining a mass constant, which was later covered with water and left to rest for 24 ± 4 h. Water was decanted, and the gravimetric method (pycnometer) and the corresponding formulas given by Ref. 19 were applied to determine the density and absorption of the material.

The granulometry and fineness modulus were also determined following the Ref. 19, the material began to be dried at a temperature of 110°C ± 5°C until a constant mass. The necessary sieves (Sieve No. 4) were selected, and mechanical sieving was conducted with 500 g of material to determine the percentage retained and the percentage that passed, which allowed the construction of a granulometric curve.

**Chemical characterization of pumice powder**

The mineral composition of the PS powder was obtained using X-ray diffraction (XRD), which is an analysis method that was conducted under a. ^20^ The pumice composition was obtained by performing X-ray diffractometry (XRD) and fineness modulus determination by dry sieving.

The SF replacement percentages for the preparation of the test tubes were defined by a bibliographic review in which it was verified that the percentages are by weight depending on the binder and vary from 2% to 10%. For this area in the present study, it was proposed that the SF replacement rates be 2.5%, 5%, 7.5%, and 10%.

Once the resistance factors and SF percentages were defined, the total volume of geopolymer used for each dosage was established. In this case, a total of six 50 mm cubes and six 40 × 40 × 160 mm beams were necessary, giving a volume of 750 (cm ^3^) and 1536 (cm ^3^), respectively, and an average density of 2200 (kg/m ^3^) obtained ^21^ was taken as a reference and a waste of 5% was considered.

Table 5 shows the dosages obtained for a variation in SF from 0% (controls) to 10% at concentrations of 8 and 12 M.

### Geopolymer mix design

To calculate the amount of materials involved in the manufacture of the geopolymer, it was necessary to identify the factors that influence the resistance of this material according to a preliminary documentary review. Table 4 lists the values adopted for each.

**Table 4.** Definition of resistance factors.

Factors	Adopted Value	References
Alkaline Concentration	8 M and 12 M	^4^
Alkaline Solution/Binder Ratio	0.75	^4^
Sodium Silicate/Sodium Hydroxide Ratio	2.5	^13^
Curing time	120 hours	^15^
Curing temperature	60°	^16^

**Table 5.** Geopolymer dosing.

SF (%)	Concentration of NaOH	Alkaline solution (Na _2_ SiO _3_ + NaOH)	Binder (kg)	PS powder (kg)	SF (kg)	Na _2_SiO _3_ (kg)	NaOH (kg)	Water weight (kg)
0	8 M	1,369	3,912	3,912	-	0.978	0.391	1,565
0	12 M	1,369	3,912	3,912	-	0.978	0.391	1,565
2.5	8 M	1,369	3,912	3,814	0.098	0.978	0.391	1,565
2.5	12 M	1,369	3,912	3,814	0.098	0.978	0.391	1,565
5.0	8 M	1,369	3,912	3,716	0.196	0.978	0.391	1,565
5.0	12 M	1,369	3,912	3,716	0.196	0.978	0.391	1,565
7.5 _	8 M	1,369	3,912	3,618	0.293	0.978	0.391	1,565
7.5	12 M	1,369	3,912	3,618	0.293	0.978	0.391	1,565
10	8 M	1,369	3,912	3,520	0.391	0.978	0.391	1,565
10	12 M	1,369	3,912	3,520	0.391	0.978	0.391	1,565

### Mixing procedure

The process began with the preparation of an alkaline solution that served as an activator for the mixture, for which the sodium hydroxide solutions were first prepared at the already defined concentrations of 8 and 12 M, and then mixed with sodium silicate.

Sodium silicate and sodium hydroxide solutions were mixed at a ratio of 2.5 for 2 min to form the activating alkaline solution, and to this, the amount of water resulting from the water/binder ratio of 0.40 was added and mixed for 2 min. On the other hand, the PS powder was integrated with the SF for 3 minutes to pour the alkaline solution and mix for another 5 minutes until a homogeneous paste was obtained.

Once the mixture was ready, 50 mm cubes and × 40×40×160 mm beams were manufactured according to the process established in Refs. 18, 19. For this, wooden molds were made, which were cleaned and greased, and the mixture was poured into them, compacted, and eliminated the empty spaces, leaving them to rest for 24 h at room temperature to later proceed with the demolding, labeling, and curing; for the latter. The specimens were placed in a Humboldt Mfg. Co for 120 h at a constant temperature of 60°C.

Once this process was completed, all the test tubes were covered with a plastic film and kept in an environment with a temperature of 18°C and a relative humidity of 50% until the testing times of 7 and 28 days were completed.

### Physical and mechanical characterization of the geopolymer

**Physical characteristics of the geopolymer**

The density of the hardened geopolymer was determined to be Ref. 22 using 50 mm cubic test tubes. It was obtained by taking the dry mass in an oven for 24 h at a temperature of 110°C ± 5°C. Subsequently, the mass of the saturated test tube was measured by immersion for 48 h. Finally, the apparent submerged mass was determined by placing the test tube in the immersion basket. Moreover, the absorption was evaluated using density data.

**Mechanical characteristics of the geopolymer**

The compressive strength of the obtained geopolymer was evaluated under the guidelines of Ref. 16, using 50 mm cubes that were compacted in two layers, for which three test tubes were necessary for each test age and dosage. The test was carried out in a Humboldt brand universal machine by applying a load on the two faces of the cube at a speed increasing in the range of 900 to 1800 N/s. In addition, the flexural resistance was determined according to Refs. 15, 23, using 40 × 40 × 160 mm beams that were supported on cylinders with their longitudinal axis perpendicular to these supports and leaving a 100 mm clearance to apply in the center of this, a load that increased at a speed of 50 ± 10 N/s. The test was carried out with a universal control machine, and both properties were determined at ages of 7 and 28 days.

## Results and discussion

### Physical and chemical characteristics of pumice powder

**Figure 5.**
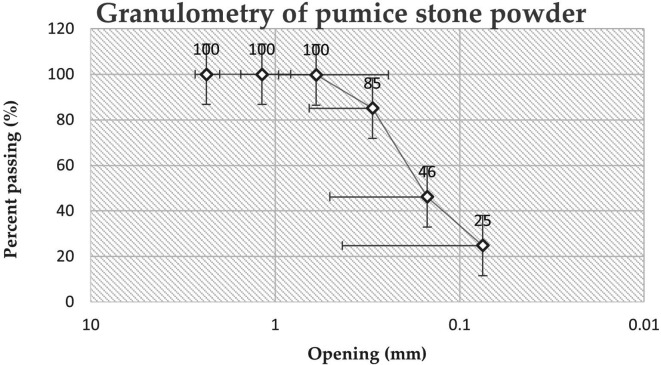
The granulometric curve of pumice stone powder.

From the results obtained in the physical characterization of the base material, the density of the PS powder was 2084.23 (kg/m ^3^) which is lower than that of other investigations, such as that of Ref. 24, where a PS powder with a density of 2500 (kg/m ^3^) is used. In addition, when comparing the granulometric curves ( Figure 5) of the materials used in the present study, only 24.78% of the material passed through the sieve. N°200 (0.075 mm), whereas in the research by Ref. 25, 57% of the material used passes through the No. 200 sieve, which indicates that the material used is thicker, which can be attributed to the grinding and sieving process of the material. It should be noted that the fineness of the base material affects the resistance of the analyzed geopolymer, as mentioned by Ref. 26.

Applying the calculation process of number 5.4 of Ref. 19, for the determination of the modulus of fineness (MF) of the PS powder, it was determined that the modulus of fineness of the PS powder is 0.69; hence, it is considered a fine material.

**Figure 6.**
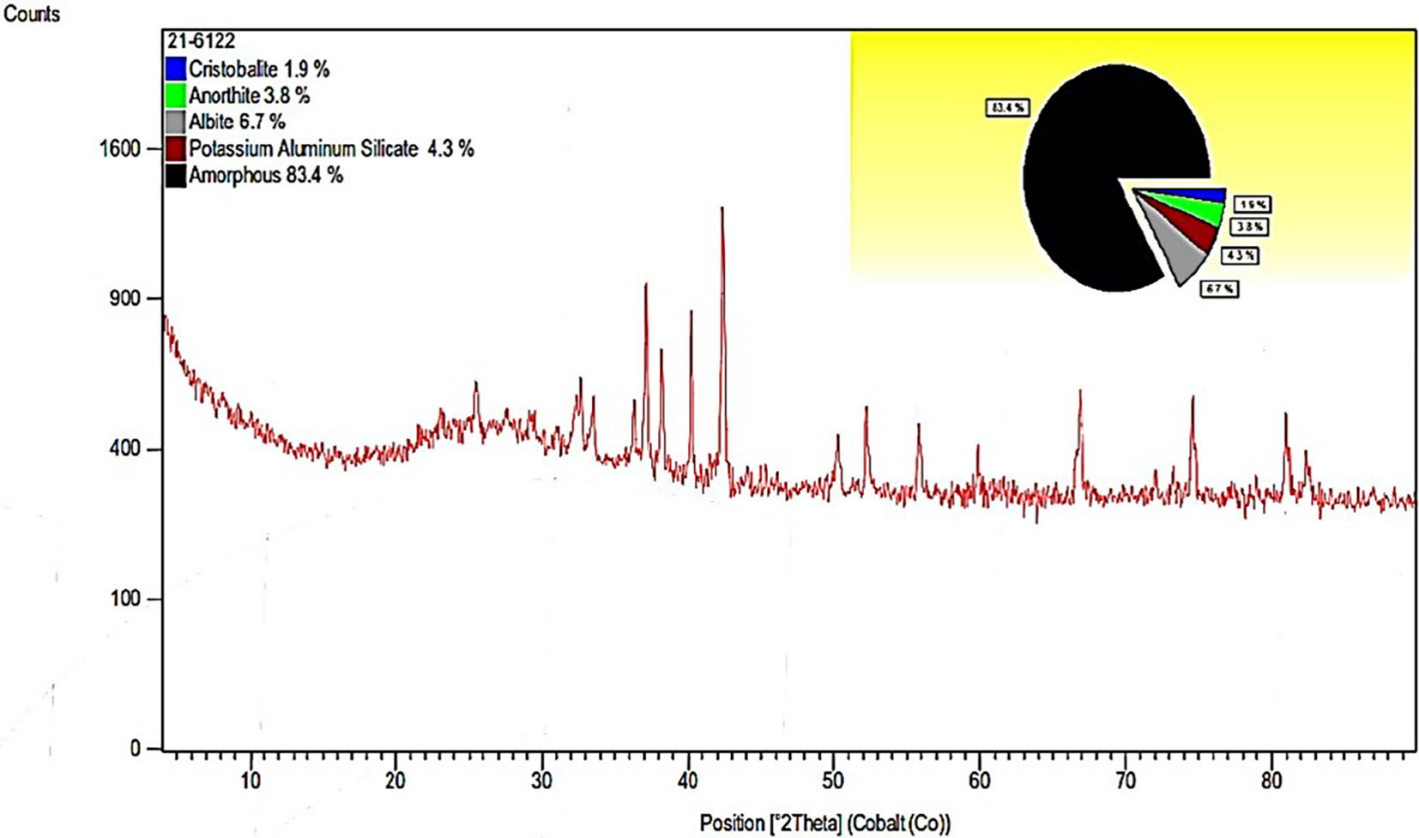
PS powder diffraction gram. ^28^

However, in the chemical characterization of the PS powder ( Figure 6 and Table 6), silicon (Si) and aluminum (Al) are common denominators in the composition of the powder. PS dust from the Cotopaxi quarry is suggested by the presence of aluminosilicates, which, according to Refs. 8, 9, 24 have the potential to be pioneers in the elaboration of geopolymers.

**Table 6.** Technical specifications of Na _2_SiO _3_.

Compound name	Chemical formula	Percentage
Cristobalite, syn	SiO _2_	1.9%
Anorthite	CaAl _2_Si _2_O _8_	3.8%
Albite, calcite, ordered	(Na, Ca) Al (Si, Al) _3_ O _8_	6.7%
Potassium aluminum silicate	K Al SiO _4_	4.3%

Table 6 summarizes the name of the compound, its chemical formula, and the percentage of PS powder.

### Physical and mechanical characteristics of the geopolymer

**Figure 7.**
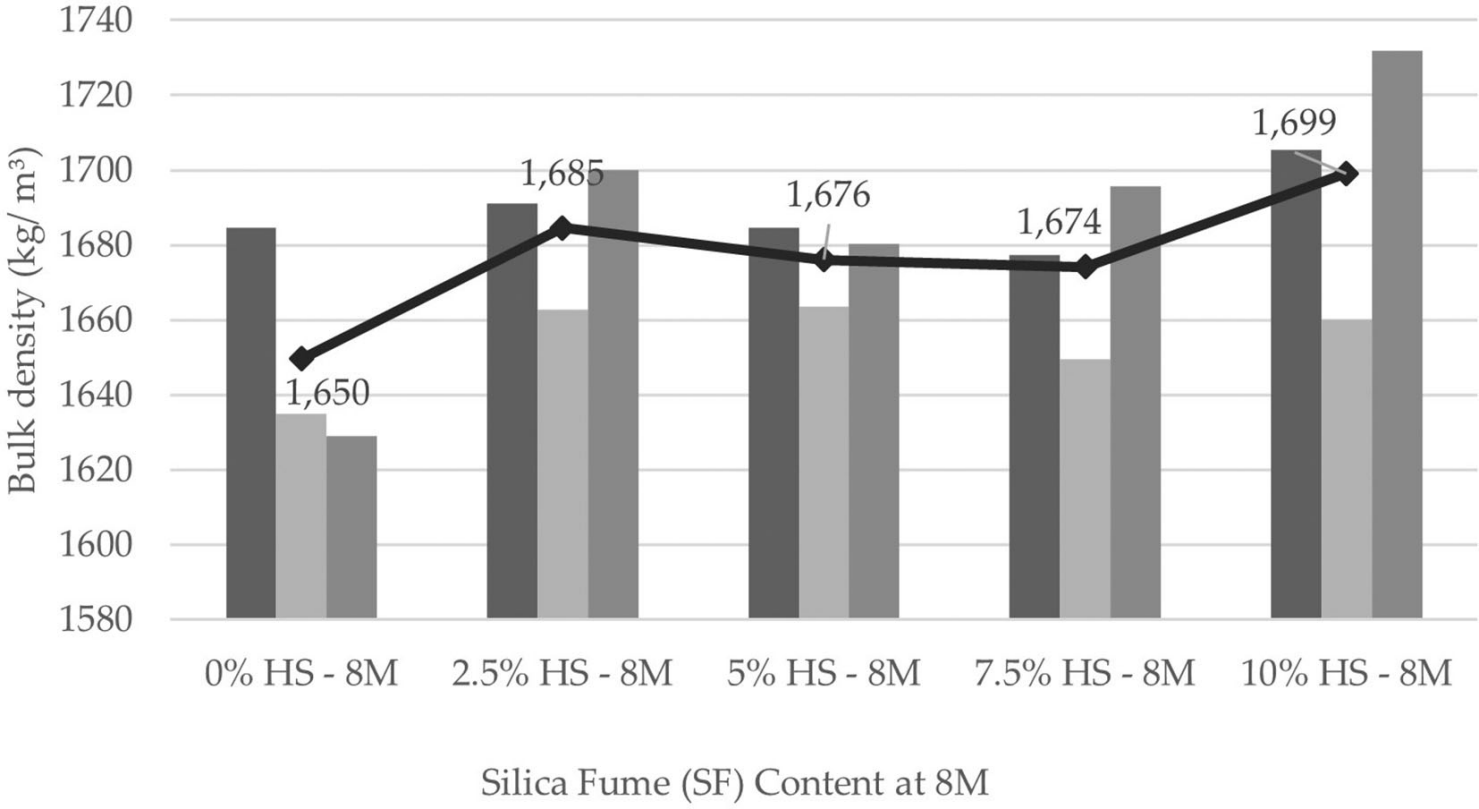
Geopolymer density at the age of 28 days with dosages at 8 M.

From the physical characterization of the geopolymer, density values ranging from 1645 (kg/m ^3^) to 1726 (kg/m ^3^) 3 were obtained ( Figure 7). This material turned out to be 25% lighter than the maximum density of 2200 kg/m ^3^ obtained by other authors as Ref. 21 of 2200 (kg/m ^3^).

The obtained geopolymer presented an average absorption percentage of 30.79%; that is, it presented many pores, and in the presence of water, its weight increased.

**Figure 8.**
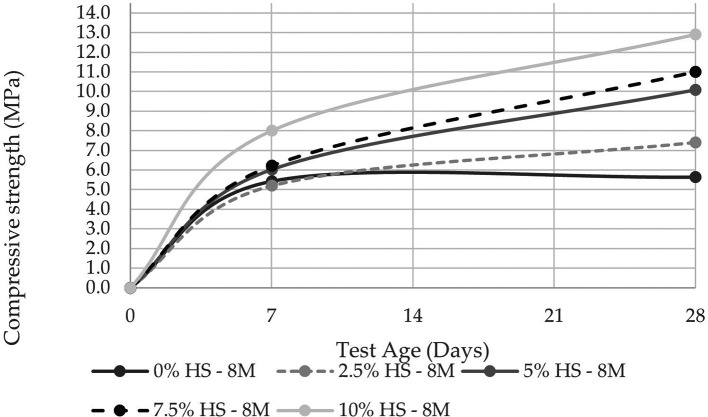
Compressive strength curves at different % of SF with 8 M.

**Figure 9.**
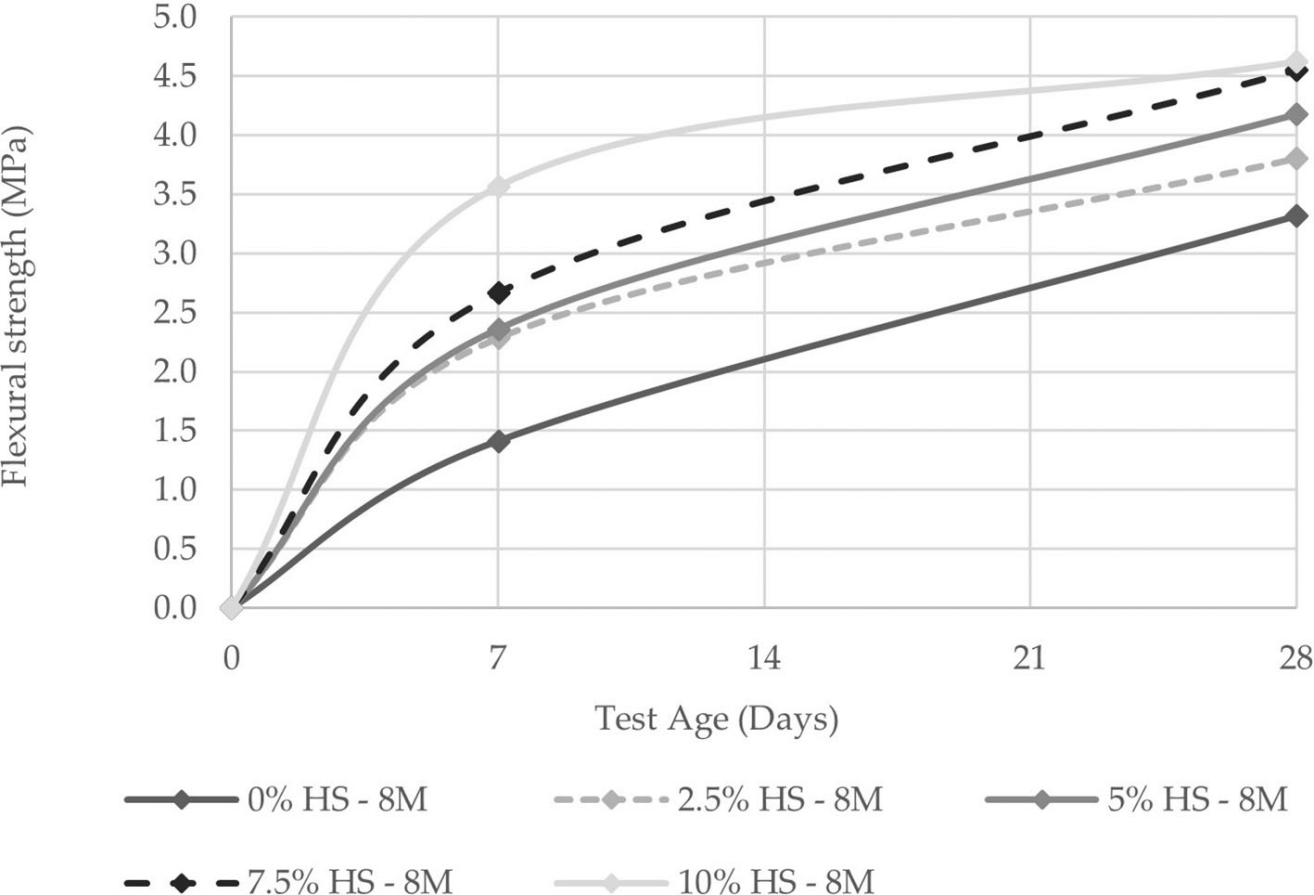
Flexural strength curves at different % of SF with 8 M.

The compressive strength was evaluated at 7 and 28 d in two molarities, one of 8 M and the other of 12 M, the latter being the concentration that allows reaching a compressive strength of 14.10 MPa at 10% SF replacement (see Figures 8 and 9). These results agree with the study carried out by Ref. 25, from which they concluded that the compressive strength was linked to the molar concentration and that a sodium hydroxide concentration of 12 M is the most optimal. Reference 27 investigated the correlation of water-cement ratios with compressive strength; the results of the water-cement vs. binder activator ratio presented in this research show a difference in strength of around 10MPa. It could be because pumice stone is in a stage of development as a precursor material of the geopolymer; furthermore, it could be due to the conditioning for the elaboration of the precursor material (pumice stone) as its treatment was carried out manually, so the size of particle generated was considerable, with a fineness modulus equal to 0.69.

Similarly, when comparing the resistance of the control specimens versus those that have 10% SF replacement, the resistance increases as the percentage of SF replacement increases, with an increase of 56.31% for the concentration of 8 M and 47.59% for that of 12 M. This demonstrates the influence that SF has on geopolymer pastes in terms of compressive strength.

**Figure 10.**
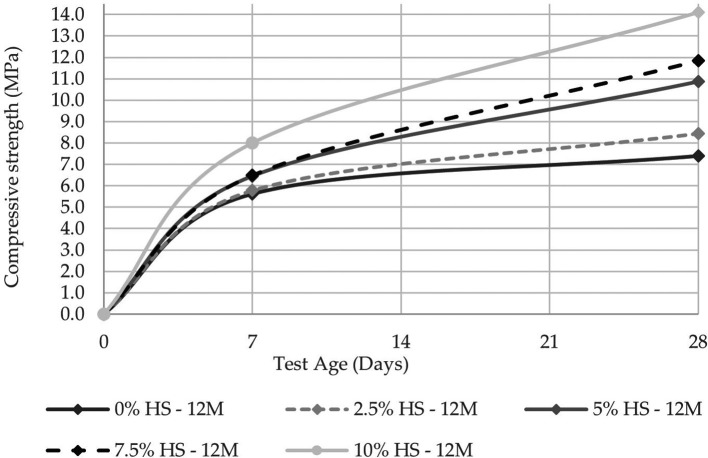
Compressive strength curves at different % of SF with 12 M.

**Figure 11.**
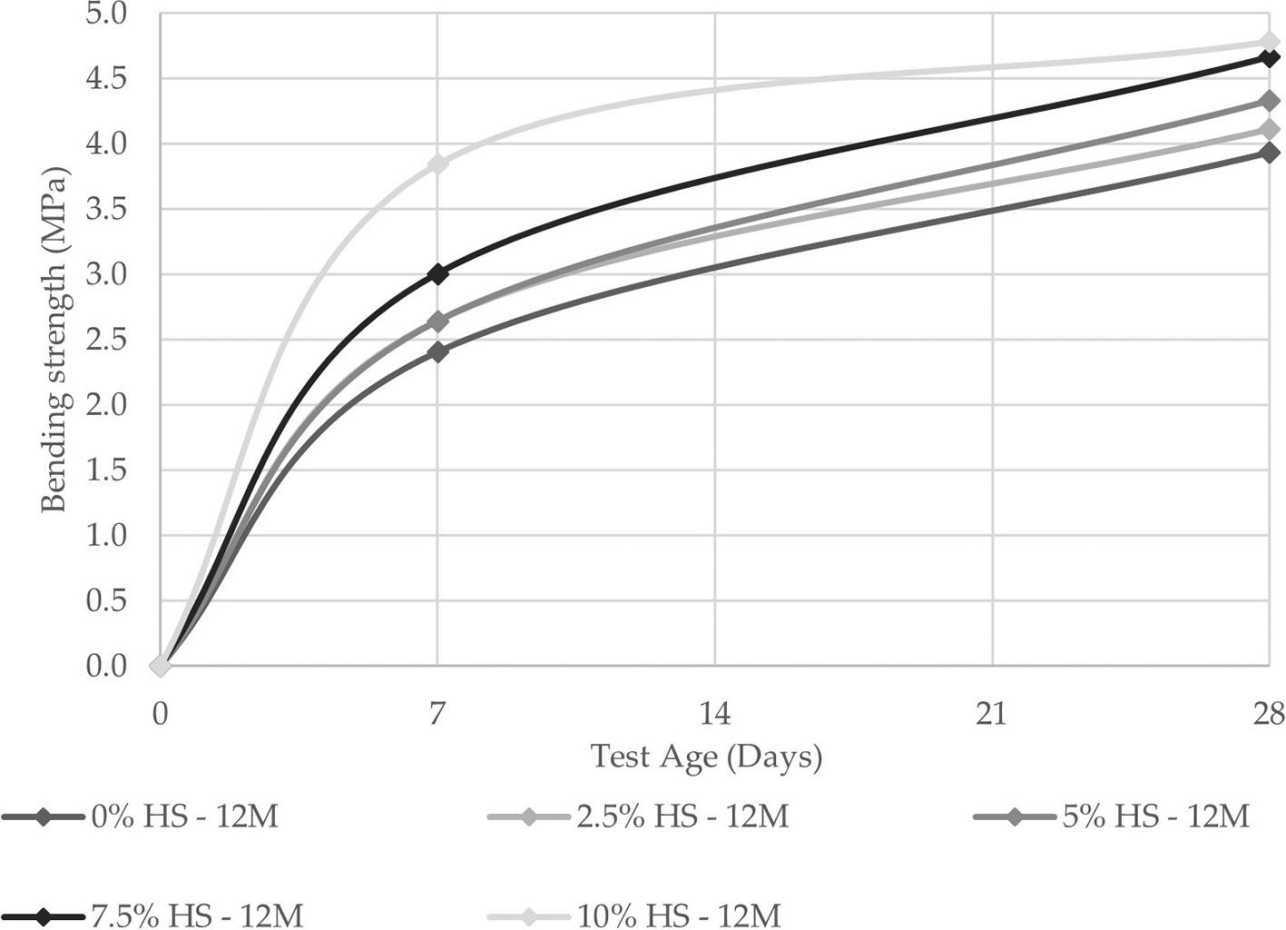
Flexural strength curves at different % SF with 12 M.

For resistance to flexure, the best results were obtained under the same conditions as the resistance to compression, while the maximum value obtained was 4.78 MPa. Figures 10 and 11 show that the increase in resistance between the control specimens and those with 10% SF replacement was 28.14% and 17.78% for the 8 M and 12 M molarities, respectively. This shows the influence of SF on geopolymer pastes when it comes to flexural strength.

**Figure 12.**
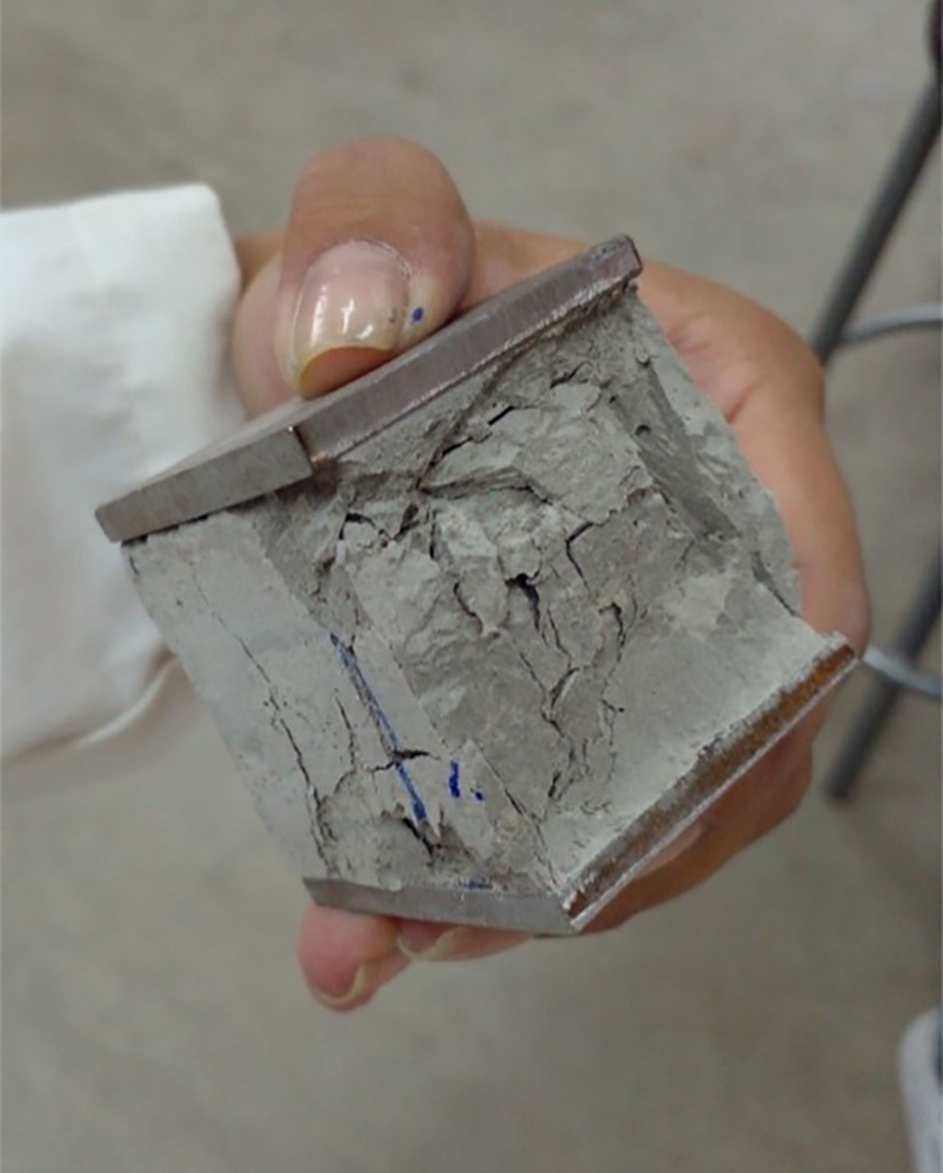
Compression test on cubic geopolymeric specimens 12 M (uniaxial compression failure).

**Figure 13.**
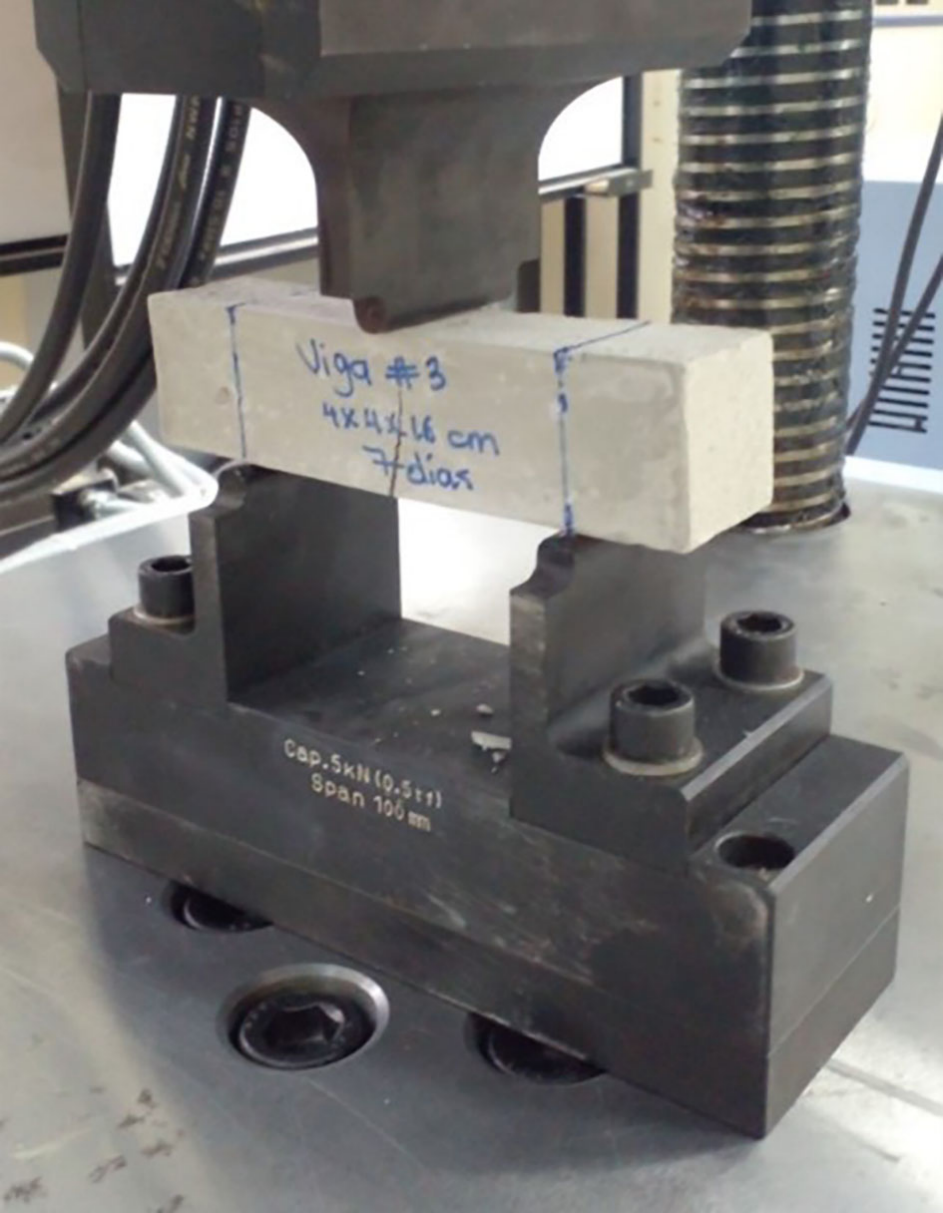
Flexure test on prismatic geopolymeric specimens 12 M (flexural failure located in the zone of maximum traction of the cross section).

Figures 12 and 13 shows compression and flexural failure for the 12M geopolymer specimens respectively.

## Conclusions

The PS powder used in the preparation of the geopolymer has a chemical composition that benefits its use as a precursor, because it is composed mostly of silicon and aluminum. Physically, it is a fine material with a particle size of less than 0.6 mm and a density of 2084.23 (kg/m ^3^).

In the design of the geopolymer dosage, several factors influence the resistance, such as the ratio of sodium silicate to sodium hydroxide, water content, temperature, curing time, molarity of sodium hydroxide and alkaline solution, and binder ratio. The latter two are the most important factors to consider in terms of environmental and economic feasibility because the excessive use of the alkaline solution is counterproductive to the contribution to reducing CO _2_ emissions, as the production of these activators requires considerable energy consumption, which represents high production costs.

The results of resistance to compression and flexure increased up to 47% and 16% respectively, this is if the test tubes with 10% replacement of SF are compared versus the control test tubes, both with 12M molarity, in this way it is evidenced that there is a directly proportional relationship between mechanical resistance and the increase in the percentage of SF replaced.

A maximum compressive strength of 14.1 MPa was reported, indicating that the geopolymer cannot be used as a structural material. However, this strength can be improved by reducing the size of the PS powder particles to a diameter of less than 0.075 mm to guarantee the dissolution and reaction of silica and aluminum, making room for polymerization of the entire material.

Regarding the physical properties of the geopolymer, the density of the specimens with SF was greater than that of the control specimens because the fineness and composition of the SF fulfill the function of filling voids, thus densifying the mixture. In addition, it contributes to the formation of the polymeric gel, which is 25% lighter if the maximum density obtained is compared with that of other authors. A high percentage of absorption caused by free water stored inside the test tubes that later evaporates during oven curing has also been reported, generating a considerable number of pores that cause a decrease in resistance.

## Data Availability

The dataset for this research has been deposited in Harvard Dataset repository and contains:
- Set of analysis results for the relevant experimental processes. Available at: Andrade Valle, Alexis, 2024, “Data - Use of pumice stone and silica fume as precursor material for the design of a geopolymer”, https://doi.org/10.7910/DVN/MHO9OV, Harvard Dataverse, V2. ^29^ Data are available under the terms of the Creative Commons Zero “No rights reserved” data waiver (CC0 1.0 Public domain dedication).
