# Supplementary material for: Use of pumice stone and silica fume as precursor material for the design of a geopolymer
Source: F1000Res. 2024 Aug 12;13:580. Originally published 2024 Jun 5. [Version 2] doi: 10.12688/f1000research.147701.2 (PMC11362735; doi:10.12688/f1000research.147701.2)
Supplement: Peer review history

# Reviewer report: Reviewer response for version 2

**DOI:** 10.5256/f1000research.169994.r359271

**Published:** 2025-02-07

**Authors:** Zrar Safari

**Affiliations:** 1 Soran University, Soran, Iraq

The manuscript presents a “Use of pumice stone and silica fume as a precursor material for the design of a geopolymer” The present study deals with using pumice as a precursor in the production of geopolymers and replacing silica fume as a partial replacement of pumice. This investigation examines geopolymer paste's strength and physical properties, specifically focusing on the influence of molarity and replacement percentage of SF on the mechanical and physical properties of combined SF-PS geopolymer. The paper subject is interesting and required minor revision as follows:

1. The research objectives should be clearly stated in the abstract, and it should be clear whether these objectives have been achieved.
2. In “Methods,” just 2.5%, 5%, and 7.5% of silica fume were mentioned; however, 10% was used but not mentioned in Methods.
3. The quality of the diagram and figures needs to be updated with better quality.
4. The chemical composition of pumice stone powder used in the study should show that Si and Al content significantly affects a geopolymer's physical and mechanical properties with pumice.
5. Add the standard specifications of the absorption and density tests.
6. The result and discussion should be rewritten in some separate subsections and discussed separately. Density, compressive strength and flexural strength, for each sub-section, discuss each parameter arranged (first effect of %SF, then effect of Molarity and effect of age of sample).
7. There are no reasons or justifications for why %SF increases strength and why 12M has higher strength.
8. The findings of the study should be compared with previous studies about a combination of SF and pumice or GGBFS and Pumice.
9. ** **English language editing is required. 
10. It is very important to provide microstructure analysis for a better understanding of the internal activation of the geopolymer paste.

Is the work clearly and accurately presented and does it cite the current literature?

Yes

If applicable, is the statistical analysis and its interpretation appropriate?

Partly

Are all the source data underlying the results available to ensure full reproducibility?

Partly

Is the study design appropriate and is the work technically sound?

Yes

Are the conclusions drawn adequately supported by the results?

Yes

Are sufficient details of methods and analysis provided to allow replication by others?

Partly

Reviewer Expertise:

Geopolymer Concrete, waste-based Geopolymers, Alkaline activated Materials

I confirm that I have read this submission and believe that I have an appropriate level of expertise to confirm that it is of an acceptable scientific standard, however I have significant reservations, as outlined above.

# Reviewer report: Reviewer response for version 2

**DOI:** 10.5256/f1000research.169994.r359270

**Published:** 2025-01-21

**Authors:** Muhammad Tariq Bashir

**Affiliations:** 1 CECOS University of IT and Emerging Sciences, INTI International University, Peshawar, Pakistan

**Comments and Suggestions for Authors**

 The manuscript entitled, “Use of pumice stone and silica fume as precursor material for the design of a geopolymer” investigates the use of pumice stone and silica fume in geopolymers, addressing sustainability by reducing reliance on traditional cement. The authors detail the characterization of materials, mix design, and testing procedures, ensuring reproducibility. However, I have few comments and suggestion to be addressed as follows:

- The discussion could delve deeper into the microstructural changes due to silica fume
- The extended curing time (120 hours) raises practical concerns for scalability and costs. Consider discussing alternative curing methods or shorter durations that might yield comparable results.
- The distinction between water in the alkaline solution and additional water is addressed, but further clarity in the methods section would be beneficial for replication.
- The authors conclude that the material is unsuitable for structural applications. Highlight potential non-structural applications.
- Strengths and limitations of the material should be more critically compared with similar research to contextualize findings. Authors can consider the following papers: [Ref 1], [Ref 2] and [Ref 3].
- Ensure consistent terminology (e.g., sodium silicate solution vs. sodium silicate) and review the text for minor grammatical issues.

**Recommendation**

 The paper contributes to research on sustainable construction materials and can be accepted after minor revisions. Addressing the mentioned comments and suggestions will further strengthen the impact and clarity of the study.

Is the work clearly and accurately presented and does it cite the current literature?

Partly

If applicable, is the statistical analysis and its interpretation appropriate?

Yes

Are all the source data underlying the results available to ensure full reproducibility?

Partly

Is the study design appropriate and is the work technically sound?

Yes

Are the conclusions drawn adequately supported by the results?

Yes

Are sufficient details of methods and analysis provided to allow replication by others?

Partly

Reviewer Expertise:

Composite Materials,  Water & Wastewater, Sustainability

I confirm that I have read this submission and believe that I have an appropriate level of expertise to confirm that it is of an acceptable scientific standard, however I have significant reservations, as outlined above.

## References

[1] : Synergistic Effects of Corn Stalk Ash and Fly Ash on the Properties of Lightweight Concrete Using Pumice Aggregate. *Iranian Journal of Science and Technology, Transactions of Civil Engineering* .2024; 10.1007/s40996-024-01491-w 10.1007/s40996-024-01491-w

[2] : Effect of Silica Fume on Compressive Strength and Water Absorption of the Portland Cement–Silica Fume Blended Mortar. *Arabian Journal for Science and Engineering* .2024;49(4) : 10.1007/s13369-023-08204-x 4803-4811 10.1007/s13369-023-08204-x

[3] : Mechanical Properties of a Sustainable Low-Carbon Geopolymer Concrete Using a Pumice-Derived Sodium Silicate Solution. *Materials (Basel)* .2024;17(8) : 10.3390/ma17081792 10.3390/ma17081792 38673149 PMC11051076

# Reviewer report: Reviewer response for version 2

**DOI:** 10.5256/f1000research.169994.r313351

**Published:** 2024-08-29

**Authors:** Dillshad K. H. Amen Bzeni

**Affiliations:** 1 Salahadin University, Erbil,, Kurdistan Region, Iraq

The author answered all the comments properly and deserves to be indexed in this Journal

Is the work clearly and accurately presented and does it cite the current literature?

Yes

If applicable, is the statistical analysis and its interpretation appropriate?

Not applicable

Are all the source data underlying the results available to ensure full reproducibility?

Yes

Is the study design appropriate and is the work technically sound?

Yes

Are the conclusions drawn adequately supported by the results?

Partly

Are sufficient details of methods and analysis provided to allow replication by others?

No

Reviewer Expertise:

NA

I confirm that I have read this submission and believe that I have an appropriate level of expertise to confirm that it is of an acceptable scientific standard.

# Reviewer report: Reviewer response for version 1

**DOI:** 10.5256/f1000research.161926.r288184

**Published:** 2024-06-28

**Authors:** Dillshad K. H. Amen Bzeni

**Affiliations:** 1 Salahadin University, Erbil,, Kurdistan Region, Iraq

The article describes using pumice combined with silica fume as a source material to make geopolymer concrete. This research is notable because it explores alternatives to normal cement by using pumice, a naturally occurring volcanic stone present in several regions. The study attempted to improve the qualities of geopolymer material by combining silica fume and pumice in varied ratios.

 The paper is written in clear and accessible English, and the technique is consistent with established research methods for data collecting and presentation. The findings are reported properly. However, the text does not provide a scientific discussion or analysis of why pumice stone powder did not give adequate strength for structural concrete. Scanning Electron Microscope (SEM) investigations would have been useful for investigating the microstructure of the geopolymer material, demonstrating how silica fume increased strength, and revealing cavities and defects in the structure.

 The specimens' 120-hour (5-day) cure duration raises questions regarding the costs and time of geopolymer manufacture. Furthermore, the article does not identify the water-to-binder ratio,  whether the water stated is part of the alkali solution or additional pure water in the  mixture.

 The article might benefit from a more in-depth examination, including earlier data on pumice stone powder, to highlight variations in the geopolymer's characteristics compared to other research findings.

Is the work clearly and accurately presented and does it cite the current literature?

Yes

If applicable, is the statistical analysis and its interpretation appropriate?

Not applicable

Are all the source data underlying the results available to ensure full reproducibility?

Yes

Is the study design appropriate and is the work technically sound?

Yes

Are the conclusions drawn adequately supported by the results?

Partly

Are sufficient details of methods and analysis provided to allow replication by others?

No

Reviewer Expertise:

Concrete technology, special types of concrete including geopolymer concrete , fiber reinforced concrete

I confirm that I have read this submission and believe that I have an appropriate level of expertise to confirm that it is of an acceptable scientific standard, however I have significant reservations, as outlined above.

# Response

**Published:** 2024-08-03

**Authors:** Andrea Zarate

**Affiliations:** Ingeniería Civil, Universidad Nacional de Chimborazo, Riobamba, Chimborazo Province, Ecuador

Dear Dillshad K. H. Amen Bzeni 

 Thank you for your comments, we have submitted an amended manuscript including your recommendations. 

**1-** **The text does not provide a scientific discussion or analysis of why pumice stone powder did not give adequate strength for structural concrete.**

 Discussion has been added to the amended manuscript, considering compressive strengths studied in other investigations. 

**2-Scanning Electron Microscope (SEM) investigations would have been useful for investigating the microstructure of the geopolymer material, demonstrating how silica fume increased strength, and revealing cavities and defects in the structure**. 

 Future research will include SEM of the geopolymer material as we are investigating the mechanical behavior of geopolymer using a variety of industrial residues 

**3-The specimens' 120-hour (5-day) cure duration raises questions regarding the costs and time of geopolymer manufacture.**

 The research has an exploratory-correlational scope, for this reason it was not intended to find the cost-benefit through its experimental implementation in the quality control laboratory of materials, in these early stages it is only intended to meet the minimum requirements to make the applicability of the geopolymer as a binder material reliable.  

**4-The article does not identify the water-to-binder ratio, whether the water stated is part of the alkali solution or additional pure water in the mixture.**

 This has been clarified in the article as the alkaline solution includes distilled water and the chemical compounds: Sodium hydroxide NaOH and Sodium silicate Na2SiO3 

**5-The article might** **benefit from a more in-depth examination, including earlier data on pumice stone powder, to highlight variations in the geopolymer's characteristics compared to other research findings**. 

 More information has been added to the amended manuscript. 

 Kind Regards,

 The authors

# Reviewer report: Reviewer response for version 1

**DOI:** 10.5256/f1000research.161926.r288182

**Published:** 2024-06-24

**Authors:** Abdullah Zeyad

**Affiliations:** 1 Jazan University,, Jazan, Saudi Arabia

Dear authors,

 Please respond to the following comments.

 1- The introduction requires expansion of previous relevant research.

 2- List all references used in this study.

 3- Provide the paper with failure models for the samples.

 4- It should highlight what the research has achieved that is consistent with the purpose of the study.

 Thanks

Is the work clearly and accurately presented and does it cite the current literature?

Yes

If applicable, is the statistical analysis and its interpretation appropriate?

Not applicable

Are all the source data underlying the results available to ensure full reproducibility?

Partly

Is the study design appropriate and is the work technically sound?

Yes

Are the conclusions drawn adequately supported by the results?

Yes

Are sufficient details of methods and analysis provided to allow replication by others?

Partly

Reviewer Expertise:

Use of pumice stone in production  geopolymer concrete

I confirm that I have read this submission and believe that I have an appropriate level of expertise to confirm that it is of an acceptable scientific standard.

# Response

**Published:** 2024-08-03

**Authors:** Andrea Zarate

**Affiliations:** Ingeniería Civil, Universidad Nacional de Chimborazo, Riobamba, Chimborazo Province, Ecuador

Dear Abdullah Zeyad 

 Thank you for your comments, we have submitted an amended manuscript including your recommendations. 

**1- The introduction requires expansion of previous relevant research.**

 Additional information has been included in the introduction. 

**2- List all references used in this study**

 The reference list has been updated including the new references used. 

**3- Provide the paper with failure models for the samples**

 Figure 12 and 13 have been included showing the failure models for the samples. 

**4- It should highlight what the research has achieved that is consistent with the purpose of the study**

 The third conclusion highlights the importance of the use of SF in geopolymeric samples by improving the mechanical behavior. 

 Kind Regards, 

 The authors
